# Possible SARS Coronavirus Transmission during Cardiopulmonary Resuscitation

**DOI:** 10.3201/eid1002.030700

**Published:** 2004-02

**Authors:** Michael D. Christian, Mona Loutfy, L. Clifford McDonald, Kenneth F. Martinez, Mariana Ofner, Tom Wong, Tamara Wallington, Wayne L. Gold, Barbara Mederski, Karen Green, Donald E. Low, on behalf of the SARS Investigation Team

**Affiliations:** * University of Toronto, Toronto, Canada; † University Health Network,Toronto, Canada; ‡ North York General Hospital, Toronto, Canada; § Centers for Disease Control and Prevention, Atlanta, Georgia, USA; ¶ Health Canada, Ottawa, Canada; # Toronto Public Health, Toronto, Canada; ** Mount Sinai Hospital Toronto, Toronto, Canada

**Keywords:** SARS virus, resuscitation, occupational health, infection control, transmission, healthcare worker

## Abstract

Infection of healthcare workers with the severe acute respiratory syndrome–associated coronavirus (SARS-CoV) is thought to occur primarily by either contact or large respiratory droplet transmission. However, infrequent healthcare worker infections occurred despite the use of contact and droplet precautions, particularly during certain aerosol-generating medical procedures. We investigated a possible cluster of SARS-CoV infections in healthcare workers who used contact and droplet precautions during attempted cardiopulmonary resuscitation of a SARS patient. Unlike previously reported instances of transmission during aerosol-generating procedures, the index case-patient was unresponsive, and the intubation procedure was performed quickly and without difficulty. However, before intubation, the patient was ventilated with a bag-valve-mask that may have contributed to aerosolization of SARS-CoV. On the basis of the results of this investigation and previous reports of SARS transmission during aerosol-generating procedures, a systematic approach to the problem is outlined, including the use of the following: 1) administrative controls, 2) environmental engineering controls, 3) personal protective equipment, and 4) quality control.

During the global spread of severe acute respiratory syndrome (SARS) (*1*–*5*), a great deal was discovered about the illness and the SARS-associated coronavirus (SARS-CoV) (*6*,*7*). SARS-CoV infection is thought to occur primarily by either contact or large respiratory droplet transmission (*3*,*8*). However, despite the use of infection control precautions and personal protective equipment designed to prevent contact and droplet transmission, episodes of SARS-CoV transmission to healthcare workers have continued to occur under certain circumstances.

Of particular concern are procedures performed on SARS patients that may aerosolize SARS-CoV and lead to limited airborne transmission or enhanced contact and droplet transmission (*9*). Such procedures include noninvasive positive pressure ventilation (BiPAP), intubation, and high-frequency oscillatory ventilation. As a result, special infection control procedures have been recommended for aerosol-generating procedures (*10*,*11*). We present the results of an investigation of the first reported transmission of SARS-CoV to healthcare workers that occurred during attempted cardiopulmonary resuscitation of a completely unresponsive SARS patient. On the basis of the results of this investigation, as well as previous reports of SARS transmission during aerosol-generating procedures, we used the continuous quality improvement framework (*12*) to suggest interventions for preventing future episodes of transmission.

## Methods

Data were collected through interviews of healthcare workers present during the attempted cardiopulmonary resuscitation where transmission of SARS-CoV was thought to have occurred. Interviews included a structured questionnaire component. Hospital and provincial policies in place at the time of the resuscitation were reviewed. The hospital patient-care environment was inspected by a team of environmental engineers and industrial hygienists. Laboratory specimens, collected with nasopharyngeal swabs, were obtained from healthcare workers with symptoms that fulfilled the SARS clinical case definition after exposure during the attempted cardiopulmonary resuscitation. These were tested by reverse transcriptase–polymerase chain reaction (RT-PCR) with primers specific for SARS-CoV (*7*). After participants gave informed consent, convalescent-phase serum was collected from all consenting healthcare workers exposed to the attempted resuscitation event as part of a larger seroprevalence study of hospital staff. For this, samples were analyzed with a commercially available indirect immunofluorescent assay (Euroimmune, Lübeck, Germany) according to the directions of the manufacturer.

In addition, a limited evaluation of the Stryker T4 Personal Protection System (Stryker Instruments, Kalamazoo, MI), worn by some of the healthcare workers involved in the resuscitation attempt, was conducted to estimate the operating parameters, including particle removal efficiency and air-flow rate. A Met One Model 227B Hand-Held Particle Counter (Met One, Inc., Grants Pass, OR) was used to count ambient particles outside and inside the hood; five replicates were collected for each condition over a 1-minute sampling period. All information was obtained as part of an ongoing joint investigation into the cause of the second phase of the Toronto SARS outbreak conducted by Toronto Public Health, Health Canada, and the Centers for Disease Control and Prevention (*13*).

### Case Report

A 67-year-old woman with a history of asthma was admitted to hospital A on May 24, 2003, with a 5 day history of fever, cough, malaise, headache, and myalgias. The patient’s mother had recently been admitted to the same hospital and died of a nosocomial pneumonia after orthopedic surgery for a fractured hip. On the basis of clinical findings and the identification of secondary infections in exposed persons, the mother’s death was retrospectively determined to be due to SARS. On admission, the patient was febrile and her chest radiograph showed left lower lobe and lingular infiltrates. Both acute-phase serologic tests and serum RT-PCR were positive for SARS-CoV (National Microbiology Laboratory, Health Canada, Toronto). She was admitted to the hospital and placed in respiratory isolation on the SARS unit. Progressive respiratory failure later developed in the patient, and within 72 hours of admission, she required 100% supplemental oxygen. On May 28, 2003, she was found to have no vital signs and cardiopulmonary resuscitation was attempted.

Nine healthcare workers participated in the resuscitation attempt. Three ward nurses (RN1–3) were the initial responders (Table). RN1 performed chest compressions while RN2 and RN3 prepared suction, oxygen, and intubation equipment. Three intensive care unit nurses (ICU-RN1–3), two respiratory therapists (RT1 and 2), and a physician (MD) also participated in the resuscitation. ICU-RN1 took over chest compressions from ward-RN1. ICU-RN2 inserted a peripheral intravenous catheter (IV) in the left foot of the patient and administered medications via the IV during the resuscitation attempt. ICU-RN3 ventilated the patient with a bag-valve-mask, without a bacterial/viral filter. RT1 performed the endotracheal intubation, which was completed in <30 seconds. No suctioning was required during or after the intubation and no respiratory secretions or other bodily substances were observed in the environment. A bacterial/viral filter was placed on the bag-valve-mask after the intubation.

**Table:** Healthcare worker exposures, personal protective equipment, and outcome^a^

Code team member	Tasks (duration of exposure)	Exposure time	Protective equipment	Symptoms (onset)	SARS serologic findings
Ward RN1	Contact before code (120 min), compressions (<5 min), assisted IV insertion (5 min), observed code (10 min), wrap body (10–15 min)	150–155 min	Gown x 2, gloves x 2, safety glasses, shoe covers, hair cover, N95 respirator	None	Refused testing
Ward RN2	Set up suction equip (5 min), charting arrest record (15 min), wrapped body (10–15 min)	30–35 min	Gown x 2, gloves x 2, safety glasses, face shield, shoe covers, hair cover, N95 respirator	None	Negative
Ward RN3	Set up oxygen equip (5 min), prepared intubation equipment (10 min), observed (5 min), wrapped body (10–15 min)	30–35 min	Gown x 2, gloves x 2, safety glasses, face shield, shoe cover, hair cover, N95 respirator	Headache, myalgia, Tmax 37.8°C (June 1)	Negative
ICU RN1	Chest compressions (10–15 min)	10–15 min	Gown x 2, gloves x 2, safety glasses, face shield, shoe cover, hair cover, N95 respirator	Headache, malise, myalgia, nausea, Tmax 38.0°C (May 31)	Indeterminate
ICU RN2	IV insertion in foot (<5 min), medication administration (10 min), application of EKG leads (<1 min)	10–15 min	Gown x 2, gloves x 2, safety glasses, face shield, shoe cover, hair cover, N95 respirator	Myalgia, malaise, SOA, Tmax 38.5°C (May 31)	Positive
ICU RN3	Ventilated patient with bag-valve-mask (5–10 min)	5–10 min	Gown x 2, gloves x 2, safety glasses, face shield, shoe cover, hair cover, N95 respirator	None	Negative
RT1	Intubated patient (<30 s), ventilated patient with bag-valve-mask (10–15 min)	10–15 min	T4 Personal Protection System, N95 respirator	None	Refused testing
RT2	Put filter on ETT and assisted RT1 (5–7 min)	5–10 min	T4 Personal Protection System, N95 respirator	None	Refused testing
MD	Chest compressions (5–7 min)	5–10 min	T4 Personal Protection System, N95 respirator	None	Refused testing

^a^SARS, severe acute respiratory syndrome; RN1, ward nurse 1; RN2, ward nurse 2; RN3, ward nurse 3; ICU-RN1, intensive care unit nurse 1; ICU-RN2, intensive care unit nurse 2; ICU-RN3, intensive care unit nurse 3; RT1, respiratory therapist 1; RT2, respiratory therapist 2; MD, physician; IV, intravenous catheter; Tmax, maximum temperature; EKG, electrocardiogram; ETT, endotracheal tube

All nurses in the room during the resuscitation were wearing protection equipment that was considered standard for routine SARS patient care at this hospital. This equipment consisted of two gowns, two sets of gloves, goggles, a full-face shield (with the exception of RN1 and RN2), shoe covers, hair cover, and NIOSH-approved N95 disposable respirators that were not fit-tested. In addition, all nurses involved in the resuscitation were experienced in working on SARS units and thus familiar with the recommended infection control policies and procedures. In contrast to the nurses, both RTs and the MD were wearing T4 Personal Protection Systems during the resuscitation. All nurses left the room immediately after the intubation and removed their protection equipment following the standard hospital protocol. Approximate exposure times are outlined in Table.

On May 31, 2003, both ICU-RN1 and ICU-RN2 had a temperature >38.0°C, myalgia, and malaise. In addition, ICU-RN1 complained of headache and nausea, and ICU-RN2 reported dyspnea. ICU-RN1 had a normal chest radiograph results, but the radiograph of ICU-RN2 showed a left lower lobe infiltrate that persisted for several days. Both RNs were admitted to the hospital for observation; their condition remained stable. RN3 reported a headache and myalgia on June 1, 2003, but her maximum temperature reached only 37.8°C. She remained in home quarantine, and her symptoms resolved without further progression. Results of RT-PCR performed on nasopharyngeal swabs from ICU-RN1 and ICU-RN2 were negative (*7*). At present, only one case (ICU-RN2) meets the World Health Organization criteria for probable SARS, one case (ICU-RN1) is under investigation, and the third (RN3) does not meet the case definition as her temperature remained <38.0°C (*14*). A review of the 48-hour period before the resuscitation did not show any other likely transmission episodes. In particular, ICU-RN2 was the charge nurse in the ICU and had little or no direct patient contact in the 48 hours before the resuscitation. Five of the nine healthcare workers involved in the resuscitation agreed to participate in serologic testing. All convalescent-phase samples were collected >30 days after the event (Table).

Evaluation of the Stryker T4 Personal Protection System indicated an average removal efficiency of 68% for particles >0.5 μm in diameter and 54% for particles >5 μm. This equates to a reduction factor (i.e., particles outside of the hood would be reduced in number by this factor) of 3.1 and 2.2, respectively.

## Discussion

This report describes the apparent transmission of SARS-CoV from a patient to healthcare workers during an attempted resuscitation. The similar symptom onset dates suggest a point source of exposure. In this case, SARS-CoV was transmitted despite healthcare workers’ wearing protection equipment designed to protect against contact and droplet transmission; no breaches in droplet protection equipment were identified, and exposure times were fairly brief. Although SARS transmission that involved intubation and BiPAP (*9*) have been reported, this episode is unique in that the patient was neither conscious nor breathing at the time of the intubation, and the intubation procedure was performed quickly and without difficulty. These factors make it less likely that transmission occurred as a direct result of the intubation procedure. Instead, it is more likely that transmission was related to events leading up to the intubation. In this case, just as in previous cases, either contact, droplet, or airborne transmission might have occurred.

Direct and indirect contact are the most common forms of transmission for most nosocomial pathogens; transmission between patients or from patient to healthcare worker usually follows contamination of the healthcare workers’ hands after touching either the patient or a fomite that came into direct contact with the patient. Large aerosol droplets (i.e., >10 μm) can, in addition to contaminating both animate and inanimate surfaces in close range of the patient, travel short distances through the air and make direct contact with the exposed mucous membranes of healthcare workers or other patients.

In contrast, airborne transmission is mediated by respiratory aerosols. These aerosols of infectious organisms contain droplet nuclei <10 μm in size and, depending upon their size within this range as well as ambient environmental conditions, can float on air currents and remain airborne for many hours (*15*–*18*). A large variety of viruses (*16*,*19*–*27*) are transmissible through both contact and airborne modes. Often, investigation of the epidemiology of nosocomial viral infections, establishes the occurrence of airborne transmission (*15*).

Two explanations may account for the transmission observed in this case: 1) an unrecognized breach in contact and droplet precautions occurred, or 2) an airborne viral load was great enough to overwhelm the protection offered by droplet precautions, including non–fit-tested N95 disposable respirators. If the last form of transmission was responsible, airborne virus may have been generated by the coughing patient (*16*) before her cardiopulmonary arrest or due to a “cough-like” force produced by the airway pressures created during asynchronous chest compressions and ventilations using the bag-valve-mask (*28*).

Regardless of the exact mode of transmission in this case, several lessons were learned through our investigation that may help reduce the risk of transmission to healthcare workers. A systematic approach to this problem is outlined considering the following framework: 1) administrative controls, 2) environmental engineering, 3) protection equipment, and 4) quality control.

### Administrative Controls

Policies and protocols for emergency resuscitation involving patients known to have or suspected of having SARS should include 1) description of the roles and responsibilities of healthcare workers responding to the emergency, 2) mechanisms to alert responders that the emergency involves a potentially contagious patient (e.g., announcing the code as an “isolation code blue”), 3) steps to limit the number of healthcare workers involved to minimize potential exposures, 4) plans for having auxiliary staff staged in a safe area where they can be easily called on if needed but otherwise preventing unnecessary exposure, 5) plans for safe disposal and cleaning of equipment used during the emergency response, and 6) procedures for disposition of the patient after the emergency, either to the ICU if resuscitation is successful or the morgue if unsuccessful.

Policies must be developed that consider all high-risk exposures or emergency situations and not just individual procedures. Policies that are too focused are of little value in dealing with the hundreds of unforeseeable possible situations that may arise. Conversely, policies that educate healthcare workers to assess the risks of a task and empower them to take appropriate protective action will be more effective. These policies should be crafted at each healthcare facility by a team that involves key stakeholders, including persons involved in the clinical response along with infection control practitioners and infectious disease experts.

It is also important to minimize the chance that a patient will suffer unwitnessed cardiopulmonary arrest or require emergency intubation on a SARS unit. Prevention of these events will involve two changes in policy. The first is to recognize that isolation wards cannot be staffed with the same nurse-to-patient ratio as a regular ward. Care of patients in isolation is more time intensive due to both the physical barriers (e.g., anterooms, doors kept closed at all times) and the required use of protection equipment. The nurse-to-patient ratio on the SARS ward at the time of the arrest was between 1:4 and 1:5; a more ideal ratio might be 1:2 or 1:3. It is also necessary to have a lower threshold for transferring patients to a higher acuity setting (i.e., ICU or stepdown unit) when they first begin to show signs of a clinical deterioration. To enable this, all patients on a SARS unit should have measurement of vital signs along with pulse oximetry at a minimum of every 4 hours. Should their oxygen saturation drop below 92% on room air one should administer oxygen through nasal prongs 1–4 L per minute to maintain saturation >92%, and increase vital signs/pulse oximetry to every 2 hours. If the patient subsequently requires oxygen through nasal prongs at >4 L per minute the responsible physician should be notified and increase vital signs or pulse oximetry to every 1 hour. Finally, if the patient requires supplemental oxygen of >40% to maintain saturation >92%, the patient should be transferred to the intensive care unit and undergo elective intubation in a controlled manner. This later policy has worked well in other SARS units, as well as in hospital A after it was implemented by one of the authors (M.L.) after this cluster.

Finally, policies should be developed to address the appropriateness and application of advanced cardiac life support for patients suffering cardiopulmonary arrest on a SARS ward. Many considerations must enter into any such discussion, including the usefulness and outcome of resuscitation efforts, particularly in unwitnessed arrests (*29*–*31*). Despite even the most well-planned and well-written policies, if healthcare workers are not trained in proper infection control practices, SARS will continue to be transmitted. Staff must be trained in both the application of policies as well as the use of protection equipment. In addition to education, practice is also important; for example, consideration should be given to staging one or more “mock SARS code blue” events.

### Environmental Controls

The second line of defense against the transmission of SARS is environmental engineering controls. These consist of physical engineering elements such as negative pressure rooms, dilution ventilation, high-efficiency particulate air filtration, ultraviolet lights, and scavenging devices. The primary goal of environmental engineering processes is to contain the infectious agent in a limited area and to minimize or rapidly decrease the viral load in the environment so that in the event of a breach in infection control process or protection equipment, the chance of healthcare workers or other patients becoming infected is minimized. In this case, a breach occurred in source control; the initial bag-valve-mask used in the resuscitation did not have a viral/bacterial filter on the exhaust. This breach may have resulted in “uncontrolled” release of aerosolized virus into the environment. However, previous studies with coxsackie virus showed that little or no virus is detectable in expired air, only in respiratory aerosols and droplets from coughing or sneezing (*16*,*21*).

### Personal Protective Equipment

The final line of protection against occupational exposure is protection equipment. The use of N95 respirators offers a level of protection against airborne transmission of SARS. However, for any form of respiratory protection to perform at the level of its full potential, it must be properly fitted to provide an adequate seal. The N95 disposable respirators used by healthcare workers in this instance were not fit-tested to ensure an adequate seal. Thus the exact level of protection afforded by the N95 respirators for each person in this case is unknown. Nonetheless, a higher level of respiratory protection should be considered in environments with a potentially very high SARS-CoV load, such as that associated with aerosol-generating procedures

**Figure 1:**
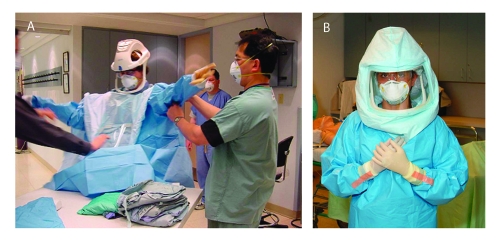
A, T4 Stryker suit being applied with aid of assistants. Healthcare worker in T4 Stryker suit. Photos provided by Randy Wax and Laurie Mazrik, Ontario Provincial SARS Biohazard Education Team.

As a result of the transmission of SARS Co-V during aerosol-generating procedures, some hospitals in Ontario, Canada, have adopted use of the T4 Personal Protective System (Stryker Instruments) (Figure 1). This system was originally designed to maintain a highly sterile field during surgery to prevent operative site infections.

As a form of protection equipment, this system has both advantages and disadvantages. The primary advantage is that the entire body of the healthcare worker is covered, providing a high level of droplet protection. The primary disadvantage of the T4 is the length of time required to put one on during an emergency. In the emergency resuscitation described in this report, the delay in certain rescuers responding was due to the time required to put on the T4. This resulted in the need for a second code blue to be announced for the same patient, which drew additional personnel to the code and thus increased the number of healthcare workers exposed to SARS.

The healthcare worker must also be attentive to avoid contamination when removing the T4. Moreover, the airborne reduction factors of 3.1, for particles >0.5 μm in diameter, and 2.2 for particles >5 μm were less than the protection factor of 10 that is assigned (i.e., minimum expected in practice) for a fit-tested, disposable N95 respirator. However, a disposable N95 is commonly worn under the T4 used in Ontario hospitals, suggesting the respiratory protection afforded healthcare workers using the T4 would be greater.

**Figure 2:**
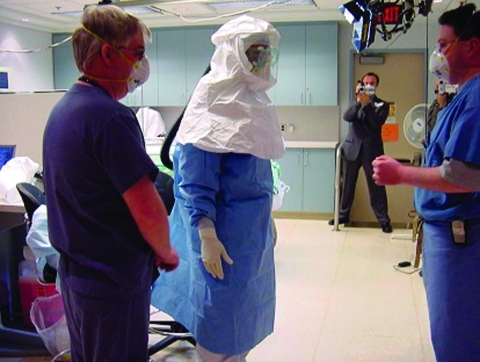
Healthcare worker wearing powered air-purifying respirators for demonstration. Photos provided by Randy Wax and Laurie Mazrik, Ontario Provincial SARS Biohazard Education Team.

The powered air-purifying respirators (PAPRs) most commonly used in healthcare settings have a disposable full hood with face shield covering the healthcare worker’s upper body (Figure 2). This device provides a higher level of protection against airborne infectious agents (any PAPR equipped with a hood or helmet with any type of particulate air filter has an assigned protection factor of 25 [*32*]), and it may be faster and easier to apply in an emergency situation. Finally, ensuring that a hospital has adequate protection against airborne diseases, even if not absolutely required for SARS, will ensure that staff are prepared to deal with future emerging infectious diseases or bioterrorism events that could involve airborne agents.

Regardless of what device (T4 versus PAPR) is used in an institution for potentially aerosol generating procedures, it is essential that they are distributed throughout the hospital in areas where they are most likely to be required by primary responders in an emergency situation as opposed to a central area where teams must wait for them to be brought to the emergency. In addition, extra protection equipment should be included as part of any “crash cart” used by the responding code team.

### Quality Control

Although there is a tendency to focus only on high-tech forms of protection equipment, it is important not to forget the basics of infection control procedures such as glove changing and hand hygiene. Healthcare workers must remain vigilant about not only protecting themselves from SARS transmission but also protecting against patient-to-patient transmission. As was found in the second phase of the SARS outbreak in Toronto (*13*), one of the best ways to prevent healthcare worker infections is to ensure that no sustained transmission of SARS occurs within the patient population, which may act as a reservoir of infection.

After developing good policies and training staff who are rehearsed for emergencies and provided with appropriate protection equipment, the last step is to ensure ongoing adherence to the standards set. This adherence is achieved through quality control. Without an effective quality control program in place, lapses in infection control procedures will occur, particularly as healthcare workers become fatigued during a prolonged outbreak.

A variety of quality control methods can be implemented, including administrative checks to ensure equipment is in good repair, policies are current, and training materials are up to date. Another quality control practice often used by emergency services personnel dealing with hazardous situations is the “buddy system.” In this system, healthcare workers always work in teams on SARS units with each person being responsible for double checking to make sure that their partner is wearing appropriate equipment and following correct infection control practices before entering a patient’s room. Finally, a process should be in place to review responses to emergencies after they have occurred to learn from the experience and facilitate continuous quality improvement.

## Conclusion

SARS has increased the medical community’s awareness of issues related to occupational health and safety. It has also highlighted the importance of infection control programs and practices. A systematic approach, including administrative controls, environmental engineering, protection equipment, and quality control, is advocated to prevent future SARS-CoV transmission to healthcare workers.
